# Reactivity and Synthetic Applications of 4,5-Dicyanopyridazine: An Overview †

**DOI:** 10.3390/molecules15031722

**Published:** 2010-03-12

**Authors:** Renzo Alfini, Marco Cecchi, Donatella Giomi

**Affiliations:** Laboratorio di Progettazione, Sintesi e Studio di Eterocicli Biologicamente Attivi (HeteroBioLab), Dipartimento di Chimica, Università di Firenze, Polo Scientifico e Tecnologico, Via della Lastruccia 3/13, I-50019 Sesto Fiorentino, Italy; E-Mail: renzo.alfini@unifi.it (R.A.)

**Keywords:** pyridazine derivatives, Hetero Diels-Alder reactions, nucleophilic aromatic substitutions, cage compounds, phthalonitriles

## Abstract

Despite the poor reputation of electron-deficient pyridazines in intermolecular Hetero Diels-Alder (HDA) reactions, 4,5-dicyanopyridazine (DCP) showed a surprising reactivity as a *heterocyclic azadiene* in inverse electron-demand HDA processes with different dienophiles. The use of alkenes, alkynes and enamines as 2π electron counterparts afforded dicyanocyclohexa-1,3-dienes and substituted phthalonitriles, respectively, while the use of suitable bis-dienophiles provides a general strategy for the one-pot synthesis of polycyclic carbo- and hetero-cage systems through pericyclic three-step homodomino processes. HDA reactions with heterocyclic dienophiles allowed direct benzoannelation: in particular, pyrrole and indole derivatives were converted to dicyano-indoles and -carbazoles. In addition an unprecedented reactivity of DCP as a very reactive *heterocyclic electrophile* at the C-4 carbon was also evidenced: by changing the experimental conditions, cyanopyrrolyl- and cyanoindolyl-pyridazines were obtained through reactions of pyrrole and indole systems as carbon nucleophiles in formal S_N_Ar2 processes where a CN group of DCP acts as leaving group. Thus, careful control of the reaction conditions allows exploitation of both pathways for the synthesis of different classes of heterocyclic derivatives.

## 1. Introduction

Pyridazine and its benzo derivatives have been known since the nineteenth century, but interest in these compounds was quite limited compared to other nitrogen heterocycles, like for instance the pyrimidines, because only a few examples of naturally occurring 1,2-diazines have been reported. Anyway, in the last decades, the biological activity exhibited by many synthetic derivatives has stimulated intensive research on this heterocyclic system [1,2,3].

This review article concerns a very specific domain in particular, being focused on the reactivity and synthetic applications of 4,5-dicyanopyridazine (DCP, **1**), a pyridazine derivative first synthesized in 1968 but essentially only exploited in organic synthesis in the last fifteen years. According with the most significant aspects of reactivity of DCP, namely as *heterocyclic electron-poor azadiene* in inverse electron-demand Hetero Diels-Alder (HDA) reactions and *heterocyclic electrophile* in formal nucleophilic aromatic substitutions S_N_Ar2, this review is organized into four parts concerning the synthesis of DCP (Section 2), Hetero Diels-Alder reactions (Section 3), nucleophilic aromatic substitutions (Section 4), and synthetic applications (Section 5).

## 2. Syntheses of 4,5-Dicyanopyridazine (DCP)

**Scheme 1:**
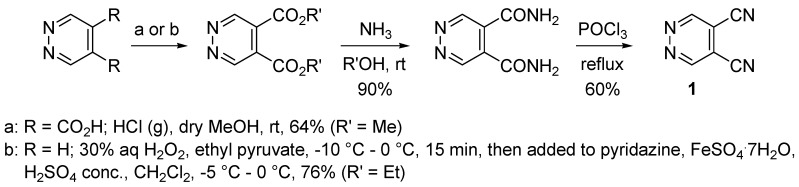
Syntheses of 4,5-Dicyanopyridazine (**1**).

The synthesis of 4,5-dicyanopyridazine (**1**) was first accomplished in 1968 by Di Stefano and Castle [4] by dehydration of pyridazine-4,5-dicarboxamide, obtained from pyridazine-4,5-dicarboxylic acid, through the corresponding dimethylpyridazine-4,5-dicarboxylate (Scheme 1, condition a). In 1985 Heinisch and Lötsch [5] reported a facile preparation of diethyl pyridazine-4,5-dicarboxylate in one step from commercially available pyridazine (Scheme 1, condition b), by radical bis-ethoxycarbonylation according to the Minisci procedure [6,7].

## 3. Hetero Diels-Alder (HDA) Reactions

Aromatic pyridazine derivatives can participate as 4π-electron components in concerted cycloadditions [1,2,3]. In particular, *N*-oxides, *N*-imides, and *N*-ylides behave as 1,3-dipoles with different dipolarophiles and, starting from the pioneering works of Neunhoeffer [8,9,10] and Jojima [11], suitably substituted pyridazines have been exploited as azadienes in inverse electron-demand HDA reactions with electron-rich or strained dienophiles or in intramolecular processes [12,13,14,15,16,17]. In this context, while the great synthetic value of 1,2,4-triazines and 1,2,4,5-tetrazines as excellent azadienes in cycloaddition processes with a variety of dienophiles has been recognized over the past decades [12,13,14,15,16,17,18,19,20,21,22,23], little attention has been devoted to 1,2-diazines, probably due to some discouraging reports suggesting their inertness towards unactivated alkynes and alkenes [15,16]. On the other hand, against all expectations, 4,5-dicyanopyridazine exhibited a surprising behaviour as a heteroaromatic azadiene in inverse electron-demand HDA reactions with different dienophiles.

### 3.1. Reactions of DCP with Dienes: A Direct Access to Carbo- and Hetero-Cage Systems

**Scheme 2:**
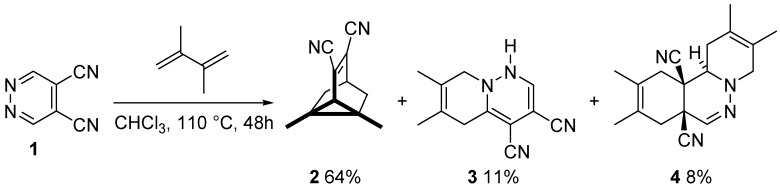
Reaction of DCP with 2,3-dimethylbuta-1,3-diene.

On the basis of a long-term study concerning Diels-Alder (DA) reactions of nitrogen heterocycles, the strongly electron-deficient DCP, structurally resembling the highly dienophilic tetracyanoethylene, was first chosen to test the almost completely disregarded applications of aromatic pyridazines as 2π-electron components in [2+4] cycloadditions. In this context, the reaction of DCP (**1**) with 2,3-dimethylbuta-1,3-diene (DMB) as 4π-electron counterpart was investigated (Scheme 2) [24]. By heating **1** with an excess of DMB in CHCl_3_ at 110 °C, in a sealed tube, only minor amounts of compounds **3** and **4** resulting from the expected [2+4] process were isolated, while the main reaction product was the tricyclic cage compound **2**, obtained in 64% yield.

**Scheme 3:**
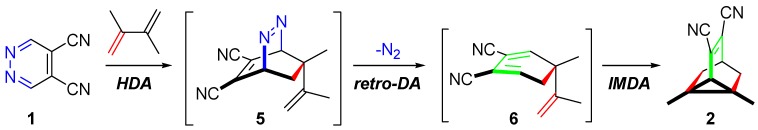
Mechanistic rationale for the formation of compound **2.**

The non-straightforward formation of **2** can be rationalized on the basis of a three-step pericyclic homodomino process [25] involving DCP as masked bis-diene [26]. A first inverse electron-demand [4+2] intermolecular HDA reaction of the electron-poor azadiene moiety of **1** on a double bond of DMB, acting as dienophile, affords the primary cycloadduct **5** able to evolve *via* retro-DA with nitrogen extrusion into the vinyl substituted cyclohexadiene intermediate **6**, then converted into the final product by an entropically assisted intramolecular Diels-Alder (IMDA) cycloaddition (Scheme 3). 

The above reaction was also performed with 4,5-dicyano-3,6-dimethylpyridazine [27]. Anyway, likely due to less favorable steric and electronic effects, the corresponding tetramethyl tricyclic derivative was isolated only operating at higher temperature and in low yield (26%) [24].

Analogous domino processes of DCP with different bis-dienophiles have been exploited for direct access to polyfunctionalized carbo- and hetero-cage compounds, often isolated in high yields as sole reaction products (Table 1, entries 1–8) [28,29]. In particular, the almost quantitative formation of the tetracyclic cage system obtained by treatment with cycloocta-1,5-diene (COD, Table 1, entry 1) allowed evaluation of the higher reactivity of DCP as an azadiene in inverse electron-demand HDA reactions with respect to other electron-poor 1,2-diazines: in fact, a previous study concerning different pyridazine derivatives reported that only the highly activated tetramethyl pyridazine-3,4,5,6-tetracarboxylate was able to react with COD, in more drastic conditions (150 °C), affording the corresponding cage system in only 19% yield [17].

**Figure 1:**
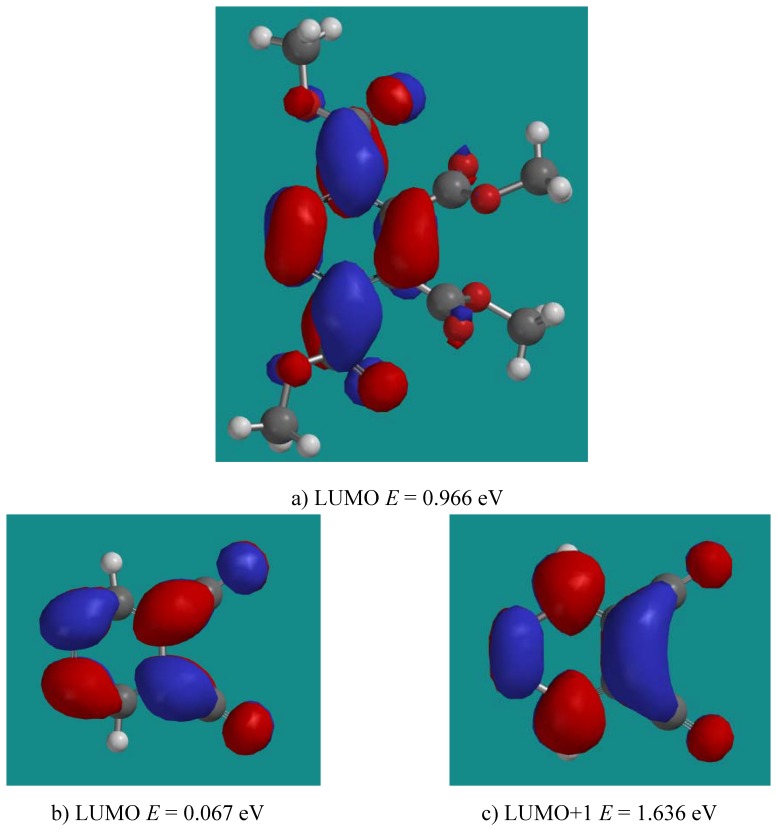
Molecular orbitals of tetramethyl pyridazine-3,4,5,6-tetracarboxylate and 4,5-dicyanopyridazine.

Although FMO calculations evidenced for DCP a LUMO of lower energy (0.067 eV, Figure 1b) with respect to the corresponding orbital of the tetraester (0.966 eV, Figure 1a), this orbital can’t participate to the HDA process due to unfavourable symmetry showing two nodes in the C-3 and C-6 positions. For this reason, DCP would be able to react with the LUMO+1 orbital, at higher energy (1.636 eV, Figure 1c) with respect to the tetraester LUMO (having a correct symmetry), and likely steric factors can be mainly invoked to justify the higher reactivity observed for the dinitrile.

A different reaction course was observed with cyclohexa-1,4-diene: a hydrogen transfer from the diene to DCP, followed by HCN elimination, is now preferred over the HDA process leading to 4-cyanopyridazine as the main reaction product (40%) [28]. Higher functionalized bis-dienophiles gave the desired cage compounds after longer reaction times and in lower yields, likely due to unfavorable steric factors associated with competitive reactions of the cyclohexadiene intermediates (Table 1, entries 9–11). Moreover, reacting with allyl acrylate, a preferential aromatization of the cyclohexadiene derivative led exclusively to the corresponding phthalonitrile (Table 1, entry 12). On the other hand, a domino reaction of DCP and diallyldimethylsilane was unsuccessful and the cage system was synthesized through a two-step process involving formation of the cyclohexadiene intermediate under milder reaction conditions (70 °C, 6 days, 66%) and subsequent IMDA reaction (110 °C, 9 days, 32%), likely hampered by the encumbering methyl groups (Table 1, entry 13). Similar steric effects caused a significant decreasing of reactivity in the reaction with myrcene, characterized by a total sitoselectivity, and with the enantiopure terpenes (*R*)-(–)-*β*-citronellene and (*R*)-(–)-linalool affording the diastereomeric tricycloundecene systems (Table 1, entries 14–16). With longer bis-dienophiles as octa-1,7-diene, nona-1,8-diene, and diallyl carbonate, the IMDA process was totally inhibited, probably due to unfavorable entropic effects and repulsive non-bonding interactions, and only cyclohexadiene derivatives were isolated (Table 1, entries 17–19).

### 3.2. Reactions of DCP with Alkenes

**Scheme 4:**
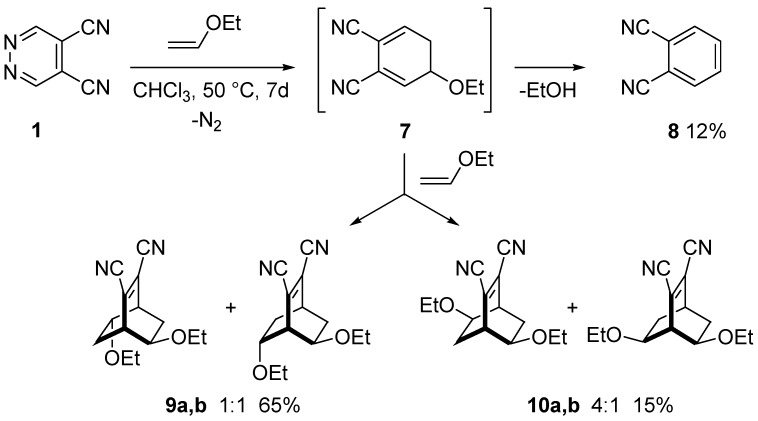
HDA reaction of **1** with ethyl vinyl ether.

4,5-Dicyanopyridazine shows an exceptional reactivity towards different 2π-electron counterparts [30]. By treatment with an excess of the activated ethyl vinyl ether in chloroform at 50 °C: the intermediate **7**, even under these mild conditions, escaped from isolation and it was converted into benzonitrile **8**, by loss of EtOH, and mainly into the *endo/syn* and *endo/anti* adducts **9a,b** and **10a,b**, isolated as 1:1 and 4:1 regioisomeric mixtures in 65 and 15% yields, respectively (Scheme 4) [31].

**Scheme 5:**
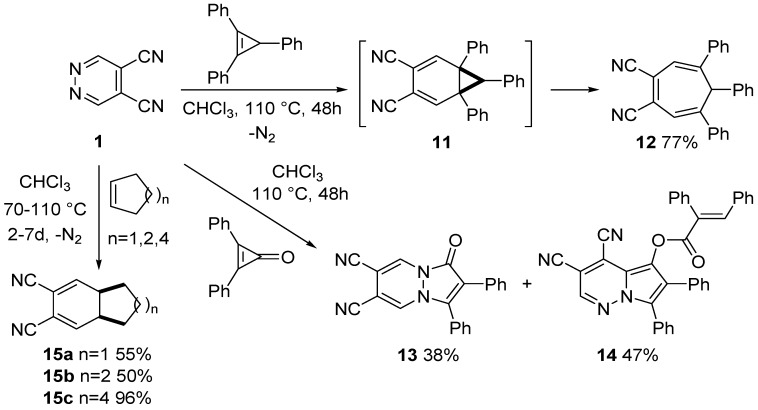
Reactions of **1** with cycloalkenes.

The reaction with the strained 1,2,3-triphenylcyclopropene in chloroform at 110 °C afforded the cycloheptatriene **12** in 77% yield through a [4+2] HDA cycloaddition followed by ring-enlargement *via* electrocyclic ring-opening of the cyclohexadiene moiety (Scheme 5). Nevertheless, DCP prefers to react as nucleophile with the strongly electrophilic diphenylcyclopropenone leading to bicyclic pyridazine derivatives **13** and **14** [31]. HDA reactions with different cycloalkenes allowed to prepare bicyclic dicyanocyclohexadienes **15a-c** (Scheme 5). With cyclopentene and cyclohexene, the yields of compounds **15a,b** were lowered due to the competing formation of bis-adducts and aromatization products [31].

**Scheme 6:**
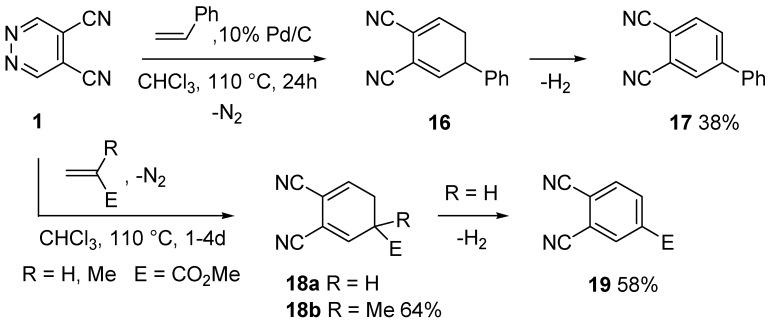
HDA reactions of **1** with linear alkenes.

Treatment of DCP with an excess of styrene at 110 °C in the presence of 10% Pd/C, to favor the aromatization of the cyclohexadiene intermediate **16**, allowed to isolate 3,4-dicyanobiphenyl **17** in 38% yield. Compound **16** was the predominant product operating at 70 °C with a stoichiometric amount of reagent but it escaped any efforts of isolation [31]. The dinitrile **1** was also able to react at 110 °C with electron-poor alkenes, as methyl acrylate and methyl methacrylate, leading to the aromatic ester **19** and the diene **18b** in 58 and 64% yields, respectively (Scheme 6) [31].

### 3.3. Reactions of DCP with Alkynes and Enamines

**Scheme 7:**
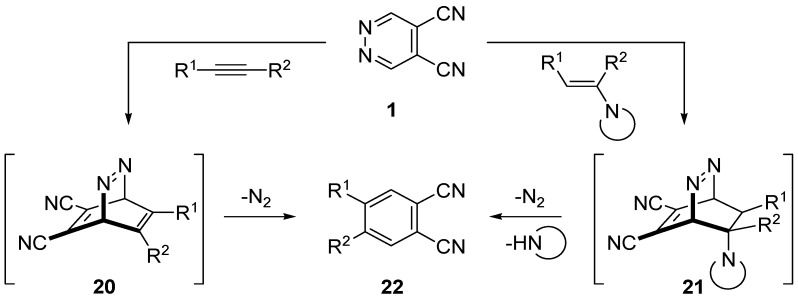
HDA reactions of **1** with alkynes and enamines.

Phthalonitriles, key building blocks for the synthesis of phthalocyanines whose importance is associated to manifold applications in different fields of material science (see Section 5), are generally obtained by multistage elaboration of aromatic precursors. On the other hand, a novel complementary direct approach to variously substituted phthalonitriles was achieved through HDA cycloadditions of DCP with alkynes and enamines (Table 2). [32]. The aromatic products **22**, isolated in satisfactory yields, come from the primary adducts **20** and **21** by nitrogen extrusion or sequential elimination of nitrogen and amine, respectively (Scheme 7).

**Scheme 8:**
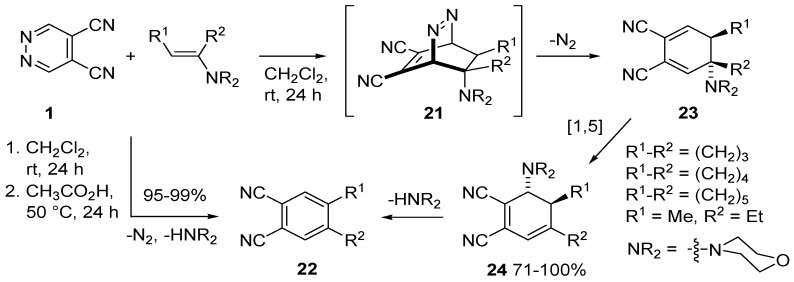
Mechanistic study of reactions of **1** with enamines.

While DCP was able to react at room temperature with the electron-rich ynamine affording the corresponding phthalonitrile derivative in 85% yield, silylacetylenes required more drastic conditions (110 °C) but allowed the isolation of the reaction products in almost quantitative yields (Table 2, entries 1–3). Reactions of scarcely activated or hindered alkynes were performed at 150 °C in xylene, for longer reaction times, leading to substituted phthalonitriles in lower yields (42–72%) (Table 2, entries 4a, 5a, 6, 7a, 8a). With the exception of highly hindered and/or less activated enamines that showed poor dienophilic properties towards **1** (Table 2, entries 5b and 8b), replacement of alkynes with the corresponding enamines allowed to achieve satisfactory yields (Table 2, entries 4b and 7b). Good results were obtained with cyclic enamines, affording condensed phthalonitriles (Table 2, entries 9a, 10a, 11a) [32]. When the reactions of DCP with cyclic enamines and (*E*)-4-(pent-2-en-3-yl)morpholine were performed in dichloromethane at room temperature for 24 hours, morpholinodicyano-cyclohexadiene derivatives **24** were obtained in high yields through a [1,5] sigmatropic rearrangement of intermediates **23** (Scheme 8). Operating with 4-(1-cyclopenten-1-yl) morpholine, it was possible to characterize the corresponding intermediate **23** *via* ^1^H and ^13^C NMR analyses.

This observation allowed an improved one-pot synthesis of the corresponding phthalonitriles **22**: in fact, by operating first at room temperature in dichloromethane for 24 hours and then, once formed compounds **24**, by addition of acetic acid and heating at 50 °C for the same time, to favor the amine elimination step, the desired products were isolated in almost quantitative yields (Table 2, entries 7c, 9b, 10b, 11b) [33].

### 3.4. Reactions of DCP with Heterocyclic Dienophiles

**Scheme 9:**
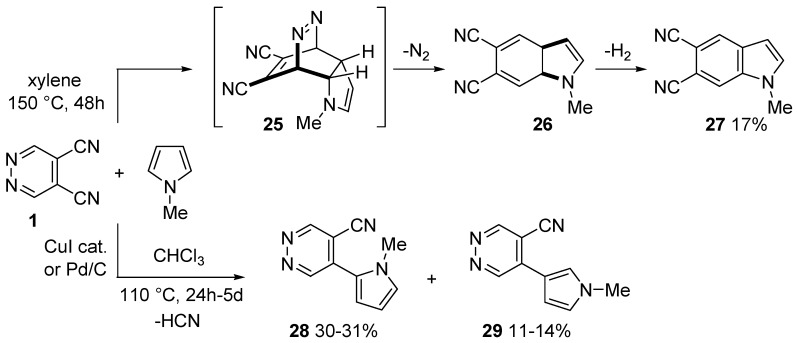
HDA reactions of DCP with *N*-methylpyrrole.

Although indole and carbazole derivatives, intensively studied for their pharmacological properties, are mainly synthesized through cyclization of suitably substituted aromatic carbocycles, alternative routes based on cycloaddition processes have also been reported [34,35,36,37,38]. In particular, in the last fifteen years electron-rich heterocycles with latent enamine functionalities, such as pyrrole and indole derivatives, have been widely exploited as 2π-electron components in inter- and intramolecular inverse electron-demand HDA reactions, mainly with 1,2,4,5-tetrazines and 1,2,4-triazines [16,20,21] for a facile access to complex indole alkaloids. Although in this context, apart from a few exceptions, 1,2-diazines played a minor role, reactions of dinitrile **1** with pyrrole and indole dienophiles were studied. HDA reactions of DCP with *N*-methylpyrrole in xylene at 150 °C afforded dicyanoindole **27** in only 17% yield, by spontaneous aromatization of intermediate **26**, coming from the primary adduct **25**. Attempts to improve this result by changing the reaction conditions were unsuccessful and operating in the presence of catalyst, as CuI or 10% Pd/C to favor the final aromatization step, compounds **28** and **29**, coming from competitive S_N_Ar2 reactions of the pyrrole derivative on the strongly electrophilic C-4 carbon of DCP (see Section 4), were obtained as main reaction products (Scheme 9) [39].

**Scheme 10:**
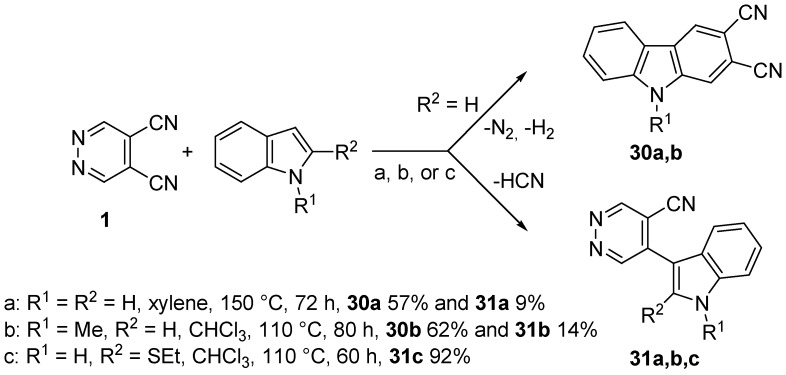
HDA reactions of DCP with indole derivatives.

Better results were achieved with the more dienophilic indole and *N*-methylindole, leading to the carbazole derivatives **30a,b** in 57 and 62% yields, respectively. Anyway, in both cases, minor amounts of substitution products **31a,b** were obtained. Moreover, while 3-acetoxy and 3-ethylthioindole were completely inert towards DCP, 2-ethylthioindole easily reacted with **1** but only compound **31c** was isolated in 92% yield (Scheme 10) [39].

**Scheme 11:**
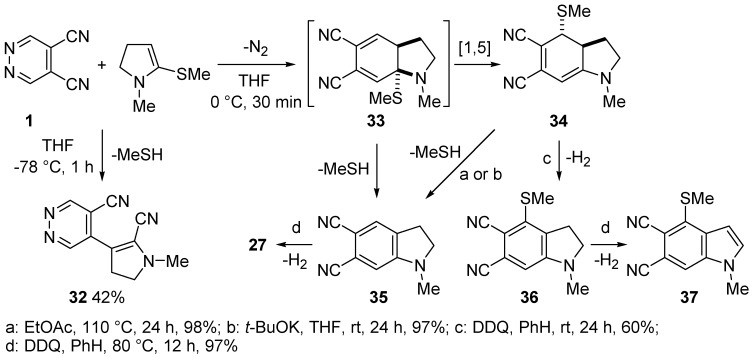
HDA reactions of DCP with 2-methylthiopyrroline.

The synthesis of indole derivatives was improved by using the non-aromatic 2-methylthiopyrroline as a strongly activated dienophile (Scheme 11).

Although at low temperature (-78 °C) the substitution pathway leading to compound **32** as predominant product is strongly preferred, by operating at 0 °C in THF for 30 minutes the bicyclic systems **34** and **35** were isolated in 50 and 23% yields, respectively [40]. The formation of **34** and **35** can be rationalized through two domino processes involving a [4+2] HDA reaction of **1** with loss of nitrogen affording the intermediate **33** and, respectively, a [1,5] sigmatropic rearrangement, as previously observed with enamines [33], or methanethiol elimination. Moreover, compound **34** is able to evolve into **35** and **36** by elimination of MeSH or H_2_. Then, this alternative methodology, now involving multi-step processes, allows to synthesize **27** in 70% overall yield, as well as the thiomethyl derivative **37**, through aromatization of **35** and **36**, respectively.

**Scheme 12:**
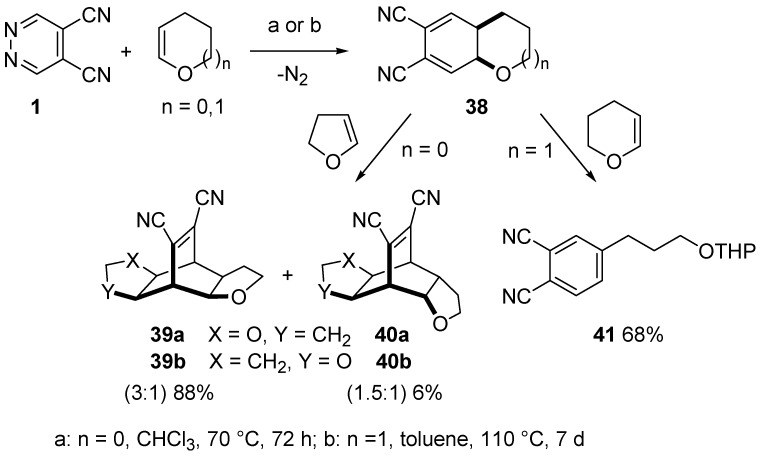
HDA reactions of DCP with dihydroheterocycles.

On the other hand, HDA reactions with heterocyclic enol ethers as dienophiles resulted in the formation of bis-adducts, as previously observed with ethyl vinyl ether (see Section 3.2). In fact, 2,3-dihydrofuran afforded the tetracyclic derivatives **39a,b,** as a 3:1 regioisomeric mixture, through *endo/anti* cycloaddition of the excess of dienophile on the cyclohexadiene intermediate **38** (n = 0), together with minor amounts of **40a,b** (1.5:1 mixture) arising from *endo/syn* or *exo/anti* approaches. The phthalonitrile **41** was the main product from the reaction of DCP and 3,4-dihydro-2*H*-pyran, obtained by ring-opening of the corresponding intermediate **38** (n = 1)and trapping with the excess of reagent (Scheme 12) [40].

## 4. Nucleophilic Aromatic Substitutions on DCP

**Scheme 13:**
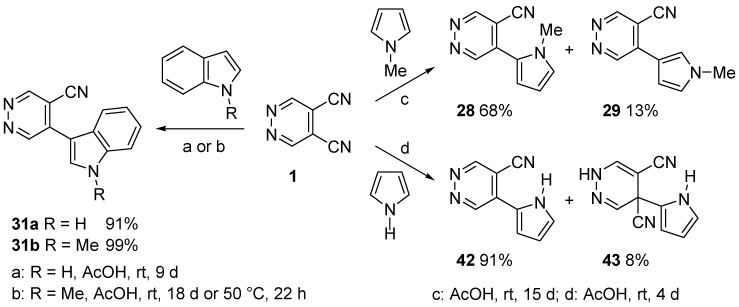
S_N_Ar2 reactions of DCP with pyrrole and indole nucleophiles.

The nucleophilic substitution of the cyano group in cyano heterocycles is quite unusual and only a few examples have been reported concerning 1,2,4-triazinecarbonitriles [41,42], quinazoline-[42] and quinazolinone-carbonitriles [43], pyrazine-2,3-dicarbonitriles [44], and pyridine-[45] and pyridinium-carbonitriles [46]. In the pyridazine series, the formal replacement of the CN group has been observed in the treatment of 4-cyano-3(2*H*)-pyridazinone or tetrazolo[1,5-*b*]pyridazine-8-carbonitrile with phenylmagnesium chloride [47], as well as in the reactions of 1-phthalazine- and 4-cinnoline-carbonitrile with Grignard reagents [48] and cinnoline-3,4-dicarbonitrile with amines [49]. Moreover, as reported in Scheme 9 and Scheme 10, the study of HDA reactions of DCP with pyrrole and indole systems allowed to isolate the corresponding substitution products **28**, **29**, and **31** [39]. On this ground, and with the aim to elucidate the behavior of the dinitrile **1** as heterocyclic electrophile in nucleophilic aromatic substitution, the above reactions were repeated in different experimental conditions. In particular, operating at room temperature (or at 50 °C) in glacial acetic acid as solvent, without any catalysts, indole and *N*-methylindole led to indolylpyridazines **31a,b** in 91 and 99% yields, respectively, while *N*-methylpyrrole gave the regioisomeric pyrrolylpyridazines **28** and **29** in 68 and 13% yields and the more reactive pyrrole afforded, after only 4 days at room temperature, derivative **42** in 91% yield, together with minor amount of dihydropyridazine **43** (8%) (Scheme 13) [50].

These results clearly show the importance of the acidity in the substitution pathway, likely involving the LUMO of protonated DCP at lower energy (-5.883 eV) with respect to the corresponding orbital of **1** (0.067 eV). Moreover, the acidic medium, through protonation of the pyridazine nitrogen, seems to play a crucial role in the HDA/S_N_Ar2 competition, in favor of the latter process.

**Scheme 14:**
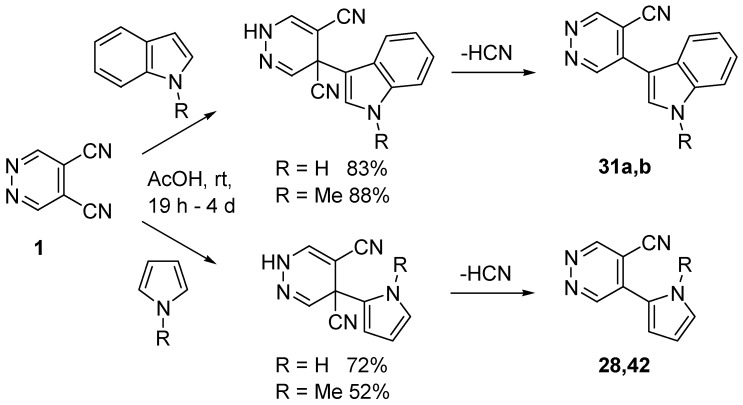
Two-step mechanism involving nucleophilic addition and elimination reactions.

The isolation of the 1,4-adduct **43**, from reaction with pyrrole, led to a mechanistic investigation of the substitution processes. Then, performing the above reactions in the same conditions but for shorter reaction times, dihydropyridazine derivatives were isolated in 52–88% yields. These intermediates were easily converted into the final products by simple stirring in AcOH at room temperature, through spontaneous HCN elimination. These data clearly show that the nucleophilic substitution products are actually the fruit of a two-step process, involving nucleophilic addition and elimination reactions (Scheme 14). To assess applications and limits of this new reactivity of DCP, reactions with different pyrrole and indole nucleophiles in acetic acid were studied.

**Scheme 15:**
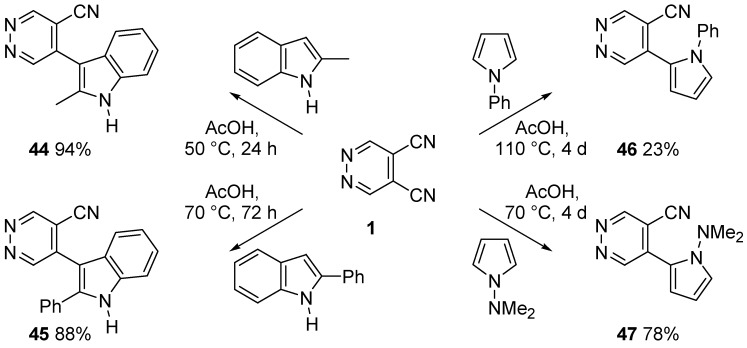
S_N_Ar2 reactions of DCP with substituted pyrrole and indole nucleophiles.

**Scheme 16:**
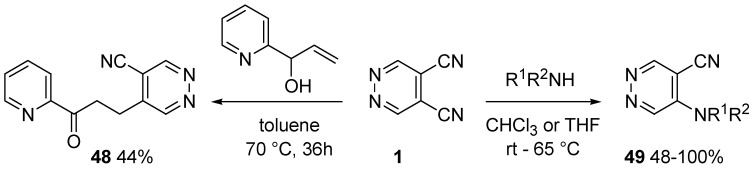
S_N_Ar2 reactions of DCP with 1-(2-pyridyl)-2-propen-1-ol and amines.

While DCP was absolutely inert towards electron-deficient-pyrrole and indole derivatives, 2-methylindole gave compound **44** in 94% yield by heating at 50 °C for 24 hours and the sterically hindered 2-phenyl derivative, although required longer reaction times and higher temperature, led to **45** in 88% yield. Reaction of **1** with the bulky *N*-phenylpyrrole was achieved in even more drastic conditions affording the pyrrolylpyridazine **46** in only 23% yield while the electron-rich *N*-(dimethylamino)pyrrole allowed to isolate **47** in 78% yield (Scheme 15) [50]. DCP was also able to react with 1-(2-pyridyl)-2-propen-1-ol, acting as C-3 carbon nucleophile, to give the polyfunctionalyzed derivative **48** by replacement of a CN group (Scheme 16) [51].

Moreover, on the basis of the reactivity observed with carbon nucleophiles, the behavior of DCP towards other nucleophilic species was also studied. In particular, some preliminary results concerning reactions with amines evidenced a facile access to aminocyanopyridazines **49**, promising for further synthetic elaborations (Scheme 16) [unpublished results].

## 5. Synthetic applications

### 5.1. Synthesis of Phthalocyanine Analogues

As previously reported, phthalocyanines have been the subject of intensive study due to their multipurpose applications as, for instance, dyes and pigments, photoconducting reagents, catalysts, deodorants, germicides, in the photodynamic therapy of cancer, and so on [52,53,54]. 

**Scheme 17:**
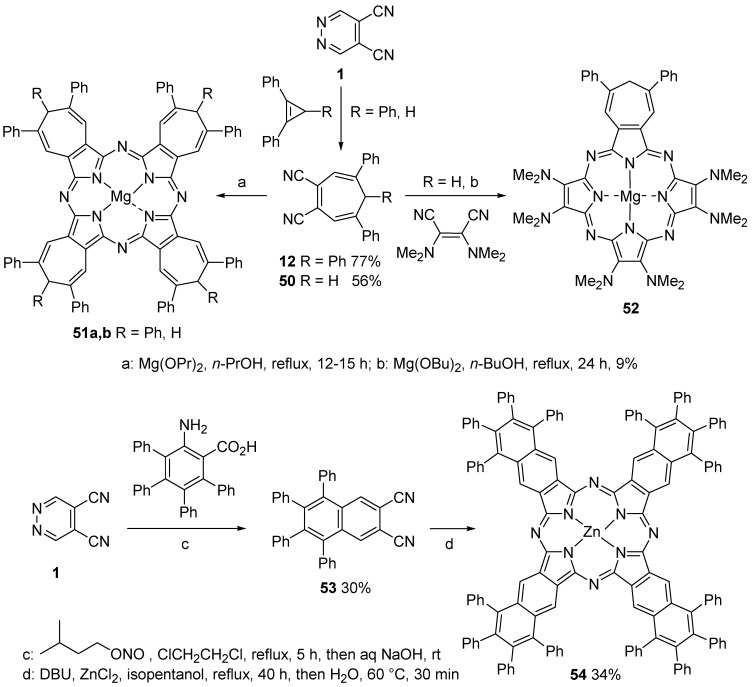
Synthesis of phthalocyanine analogues.

In this context, dicyanocycloheptatrienes **12** and **50**, obtained by HDA reactions of DCP with 1,2,3-triphenyl- and 1,2-diphenyl-cyclopropene, have been exploited for the synthesis of seven-membered carbon-ring-fused phthalocyanine analogue complexes **51a,b**, in which the π system changes during dehydrogenation/hydrogenation cycles, according with nonaromatic cycloheptatriene/aromatic tropylium cation interconversion (Scheme 17) [55].

Moreover, crossover Linstead macrocyclization of **12** and bis(dimethylamino)maleonitrile gave rise to an unsymmetrical (A_3_B) aminoporphyrazine **52** converted by hydride abstraction into a tropylium-fused aminoporphyrazine, labelled as a ‘push-pull’ macrocycle for the presence of both electron-donating and electron-withdrawing groups (Scheme 17) [56].

On the other hand, diazotization of 3,4,5,6-tetraphenylanthranilic acid in the presence of DCP allowed HDA trapping of the aryne intermediate for the synthesis of dicyanonaphthalene **53**, converted to the polyphenylated naphthalocyanine **54**. The sixteen phenyl substituents, orthogonal to the macrocyclic plane, are responsible for improved solubility and minimal intermolecular aggregation (Scheme 17) [57].

### 5.2. Synthesis of Enantiopure C_2_ Symmetric bis(2-Oxazolinyl) Cage Ligands

In the last decades, a great range of ligands containing one, two or three oxazoline rings has been successfully developed and applied in asymmetric catalysis [58]. In particular, the attention towards enantiopure *C*_2_ symmetric bis(oxazoline) (Box) ligands has increased enormously and a large number of chiral ligands with a great deal of structural diversity has been synthesized and applied in an impressive variety of metal-catalyzed processes [59]. The synthesis of oxazoline systems can be easily achieved from amino alcohols and nitrile or carboxylic acid derivatives [60] and, in particular, Bolm *et al*. described a direct reaction of dinitriles with enantiomerically pure β-amino alcohols under zinc chloride catalysis [61]. On these grounds, the cage systems obtained by domino reactions of DCP with dienes, all containing a maleonitrile moiety coming from **1**, appeared interesting substrates for the synthesis of new classes of chiral bis(oxazoline) ligands.

**Scheme 18:**
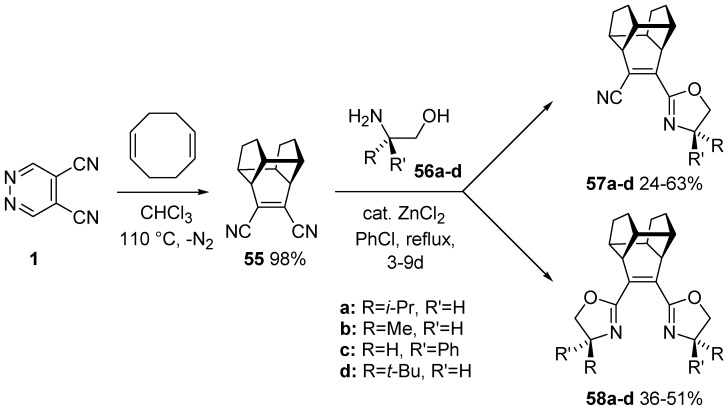
Synthesis of mono- and bis(oxazoline) ligands.

**Figure 2:**
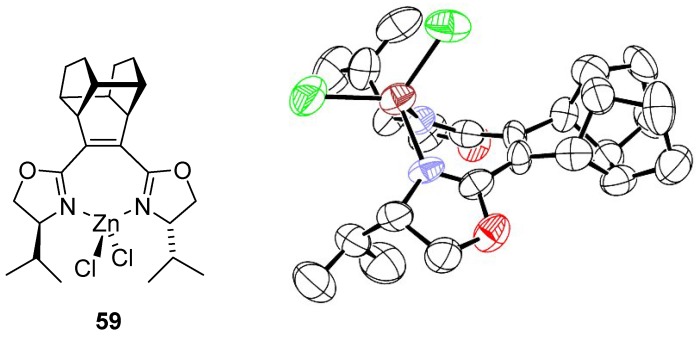
ORTEP drawing of complex **59.**

Then, the tetracyclic compound **55**, prepared almost quantitatively from reaction of **1** with COD (Table 1, entry 1), was allowed to react with enantiopure β-amino alcohols **56a-d** in anhydrous chlorobenzene under reflux, in the presence of catalytic amounts of anhydrous ZnCl_2_. Depending on the experimental conditions, different quantities of enantiomerically pure mono(2-oxazolines) **57a-d** and bis(2-oxazoline) (Cage-Box) ligands **58a-d** were isolated from the reaction mixtures. Anyway, mono(oxazolines) can be converted into the bis(oxazoline) derivatives by treatment with β-amino alcohols in the previously reported conditions (Scheme 18) [62]. A 1:1 Zn(II) complex **59** was isolated in 99% yield by treatment of **58a** with ZnCl_2_ and its molecular structure determined by single-crystal X-ray diffraction analysis (Figure 2).

## 6. Conclusions

These results clearly evidence for 4,5-dicyanopyridazine a polyvalent reactivity towards different species. In fact, although electron-deficient pyridazines, and especially symmetric derivatives [63], appeared quite inert in inverse electron-demand HDA reactions, DCP exhibited an exceptional behavior as heterocyclic azadiene able to react not only with electron-rich dienophiles but also with unactivated alkenes and alkynes, as well as electron-poor 2π electron counterparts. In addition to the synthesis of dicyanocyclohexa-1,3-dienes, a general and complementary methodology for a facile access to substituted phthalonitriles through reactions with alkynes and enamines was accomplished. Moreover, pericyclic three-step homodomino processes with bis-dienophiles allowed to realize a general strategy for the one-pot synthesis of carbo- and hetero-cage systems.

Pyrrole and indole derivatives reacted in different ways with DCP. In particular, it appears possible to modulate the reaction course by changing the experimental conditions. By simple heating at 110–150 °C in chloroform or xylene (sealed tube), HDA reactions afforded dicyano-indoles and -carbazoles *via* direct benzoannelations. On the other hand, operating in acetic acid in milder conditions (25–110 °C), DCP was able to react with the same species as a very reactive heterocyclic electrophile at C-4 carbon in formal S_N_Ar2 processes where a CN group acts as leaving group. Mechanistic investigations evidenced that cyanopyrrolyl- and cyanoindolyl-pyridazines, obtained in satisfactory yields, are the fruit of addition-elimination processes. Synthetic applications of the above reaction products were also observed in different domains.

In conclusion, the exceptional reactivity of DCP compared to other electron-poor pyridazines, mainly evidenced in HDA reactions, could be likely ascribed to the lack of any steric hindrance experienced by the approaching reagents. On this ground, the ‘flat’ 4,5-dicyanopyridazine could certainly be welcome in the fantastic ‘Flatland’ country that in 1882 the London Reverend Edwin A. Abbott described in his book.

## Figures and Tables

**Table 1:** Reactions of DCP with dienes. ^a^

Entry	Reagent	Time	Product	Yield % ^b^ [Ref.]
1	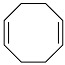	24 h	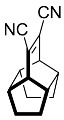	98 [28]
2	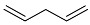	48 h	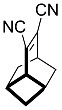	71 [28]
3	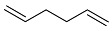	48 h	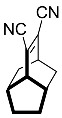	98 [28]
4	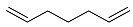	48 h	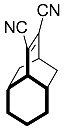	97 [28]
5	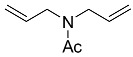	4 d	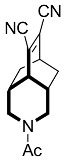	86 [28]
6	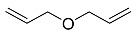	24 h	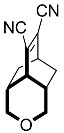	98 [28]
7	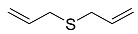	72 h	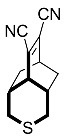	97 [28]
8	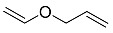	24 h	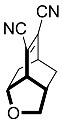	99 [29]
9	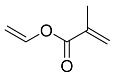	4 d	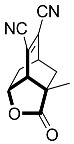	56 [29] ^c^
10	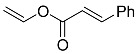	9 d	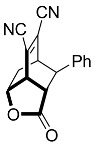	34 [29]
11	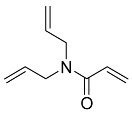	6 d	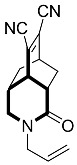	37 [29]
12	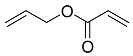	48 h	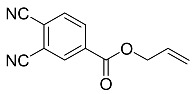	40 [29]
13*^d^*	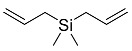	15 d	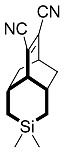	21 [29]^d^
14	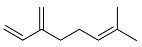	4 d	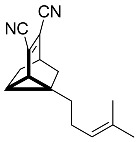	24 [29]^e^
15	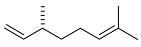	5 d	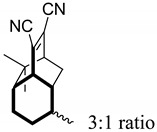	28 [29]^e^
16	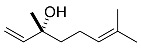	8 d	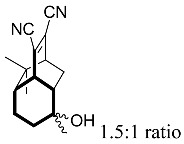	31 [29]^e^
17	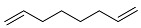	4 d	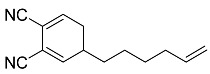	52 [29]^f^
18	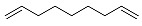	5 d	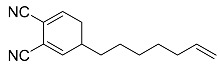	50 [29]^f^
19	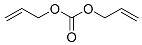	5 d	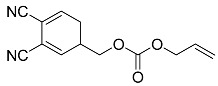	52 [29]^f^

^a^ Reactions performed in a sealed tube in chloroform at 110 °C with an excess (5 equiv) of reagent. ^b^ Isolated yields. ^c^ Phthalonitrile, formed by competitive elimination of methacrylic acid from the corresponding cyclohexadiene intermediate, was also recovered in 39% yield. ^d^ Reaction performed at 70 °C for 6 days to isolate the cyclohexadiene intermediate then converted into the cage system by heating at 110 °C for 9 days. ^e^ Competitive aromatization of the corresponding cyclohexadiene intermediates afforded minor amounts of substituted phthalonitriles. ^f^ Reactions performed with 1.1 equiv. of reagent to reduce the formation of by-products.

**Table 2:** Synthesis of phthalonitriles from reactions of DCP with alkynes and enamines. ^a^

Entry	Reagent	Conditions	Product	Yield %^b^ [Ref.]
1	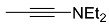	CH_2_Cl_2_, 25 °C, 19 h	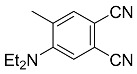	85 [32]
2	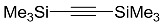	CHCl_3_, 110 °C, 48 h	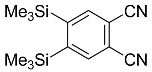	98 [32]
3	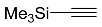	CHCl_3_, 110 °C, 8 d	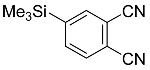	98 [32]
4a	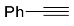	xylene, 150 °C, 24 h	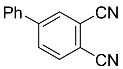	42 [32]
4b	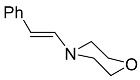	CHCl_3_, 70 °C, 24 h	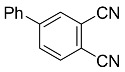	72 [32]
5a	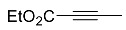	xylene, 150 °C, 5 d	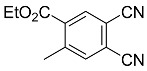	55 [32]
5b	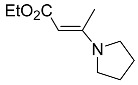	CHCl_3_, 70 °C, 24 h	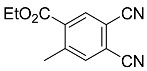	29 [32]
6	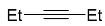	xylene, 150 °C, 10 d	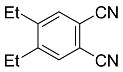	55 [32]
7a	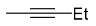	xylene, 150 °C, 18 d	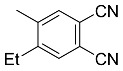	51 [32]
7b 7c	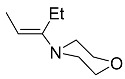	CHCl_3_, 70 °C, 7 d CH_2_Cl_2_, rt, 24 h + AcOH, 50 °C, 24 h	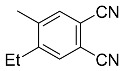	73 [32] 95 [33]
8a	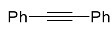	xylene, 150 °C, 21 d	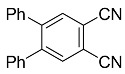	72 [32]
8b	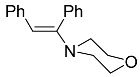	CHCl_3_, 70 °C, 48 h	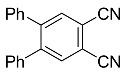	11 [32]
9a 9b	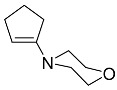	CHCl_3_, 70 °C, 24 h CH_2_Cl_2_, rt, 24 h + AcOH, 50 °C, 24 h	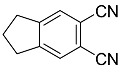	96 [32] 98 [33]
10a 10b	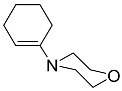	CHCl_3_, 70 °C, 6 d CH_2_Cl_2_, rt, 24 h + AcOH, 50 °C, 24 h	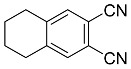	82 [32] 99 [33]
11a 11b	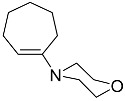	CHCl_3_, 70 °C, 7 d CH_2_Cl_2_, rt, 24 h + AcOH, 50 °C, 24 h	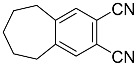	71 [32] 96 [33]

^a^ Reactions performed in a sealed tube with an excess of reagent (1.1–10 equiv). ^b^ Isolated yields.
